# Suppression of Anti-Inflammatory Mediators in Metabolic Disease May Be Driven by Overwhelming Pro-Inflammatory Drivers

**DOI:** 10.3390/nu14112360

**Published:** 2022-06-06

**Authors:** Sehar Sajid, Mohammed Gulrez Zariwala, Richard Mackenzie, Mark Turner, Theo Nell, Srikanth Bellary, Derek Renshaw

**Affiliations:** 1 Centre for Sport, Exercise and Life Sciences, Institute for Health and Wellbeing, Coventry University, Priory Street, Coventry CV1 5FB, UK; sehar.sajid13@gmail.com (S.S.); ad3759@coventry.ac.uk (M.T.); 2 Centre for Nutraceuticals, School of Life Sciences, University of Westminster, 115 New Cavendish Street, London W1W 6UW, UK; zariwam@wmin.ac.uk; 3 School of Life & Health Sciences, University of Roehampton, London SW15 4DJ, UK; richard.mackenzie@roehampton.ac.uk; 4 Centre for Cardio-Metabolic Research in Africa, Department of Physiological Sciences, Faculty of Science, Stellenbosch University Main Campus, Stellenbosch 7600, South Africa; tnell@sun.ac.za; 5 The Diabetes Centre, Birmingham Heartlands Hospital, Birmingham B9 5SS, UK; srikanth.bellary@heartofengland.nhs.uk

**Keywords:** Annexin A1, bariatric surgery, lipodystrophy, adipocytes, inflammation, FPR2/ALX receptor, AC2-26

## Abstract

Obesity is a multifactorial disease and is associated with an increased risk of developing metabolic syndrome and co-morbidities. Dysregulated expansion of the adipose tissue during obesity induces local tissue hypoxia, altered secretory profile of adipokines, cytokines and chemokines, altered profile of local tissue inflammatory cells leading to the development of low-grade chronic inflammation. Low grade chronic inflammation is considered to be the underlying mechanism that increases the risk of developing obesity associated comorbidities. The glucocorticoid induced protein annexin A1 and its *N-*terminal peptides are anti-inflammatory mediators involved in resolving inflammation. The aim of the current study was to investigate the role of annexin A1 in obesity and associated inflammation. To achieve this aim, the current study analysed data from two feasibility studies in clinical populations: (1) bariatric surgery patients (Pre- and 3 months post-surgery) and (2) Lipodystrophy patients. Plasma annexin A1 levels were increased at 3-months post-surgery compared to pre-surgery (1.2 ± 0.1 ng/mL, *n* = 19 vs. 1.6 ± 0.1 ng/mL, *n* = 9, *p* = 0.009) and positively correlated with adiponectin (*p* = 0.009, r = 0.468, *n* = 25). Plasma annexin A1 levels were decreased in patients with lipodystrophy compared to BMI matched controls (0.2 ± 0.1 ng/mL, *n* = 9 vs. 0.97 ± 0.1 ng/mL, *n* = 30, *p* = 0.008), whereas CRP levels were significantly elevated (3.3 ± 1.0 µg/mL, *n* = 9 vs. 1.4 ± 0.3 µg/mL, *n* = 31, *p* = 0.0074). The roles of annexin A1 were explored using an in vitro cell based model (SGBS cells) mimicking the inflammatory status that is observed in obesity. Acute treatment with the annexin A1 *N-*terminal peptide, AC2-26 differentially regulated gene expression (including *PPARA* (2.8 ± 0.7-fold, *p* = 0.0303, *n* = 3), *ADIPOQ* (2.0 ± 0.3-fold, *p* = 0.0073, *n* = 3), *LEP* (0.6 ± 0.2-fold, *p* = 0.0400, *n* = 3), *NAMPT* (0.4 ± 0.1-fold, *p* = 0.0039, *n* = 3) and *RETN* (0.1 ± 0.03-fold, *p* < 0.0001, *n* = 3) in mature obesogenic adipocytes indicating that annexin A1 may play a protective role in obesity and inflammation. However, this effect may be overshadowed by the continued increase in systemic inflammation associated with rapid tissue expansion in obesity.

## 1. Introduction

Obesity is a complex, multifactorial and a highly preventable disease that is considered to be the greatest epidemic of the 21st century [1]. The prevalence of obesity, which is clinically defined by having a body mass index (BMI) of ≥30 kg/m^2^ [2], has increased rapidly over the last three decades and has shown differential patterns due to gender, age, socioeconomic circumstances, lifestyle, race, geography, and time. 

Obesity arises due to excessive accumulation of fat within the adipose tissue, primarily resulting from sustained long-term positive energy balance. The adipose tissue serves two main functions: (1) storing energy and (2) regulating physiological functions including energy regulation, metabolism, and inflammation [3]. It achieves the latter by secreting a range of adipose tissue specific hormones, commonly known as adipokines and cytokines into the surrounding environment and the systemic circulation where they act in an autocrine, paracrine, and endocrine manner to regulate local and systemic metabolism and energy homeostasis [4]. While an optimum amount of adipose tissue is essential for human health, excessive adiposity is linked to multiple negative health outcomes including metabolic diseases, cardiovascular disease, and cancers. Excess adiposity is also recognized to induce low-grade chronic systemic inflammation, which is associated with the development of type 2 diabetes mellitus (T2DM) [5,6]. Thus, many anti-obesity therapies target the inflammatory status in the hope of resolving inflammation thereby, limiting the development of obesity associated metabolic comorbidities, rather than solely inducing weight loss [7].

Annexin A1 (ANXA1) is an endogenous glucocorticoid-induced protein, which is known to modulate a number of systemic anti-inflammatory processes including reducing eicosanoid synthesis, leukocyte transmigration and in the resolution of inflammation via macrophage mediated phagocytosis of neutrophils [8,9,10]. The vast range of anti-inflammatory/pro-resolving effects of ANXA1 and its peptides have shown to have significant effects in resolving inflammation in several disease models including, inflammatory bowel disease and arthritis [11,12]. It acts in an autocrine, paracrine, and endocrine manner and, exerts its effects via binding to the formyl peptide receptor (FPR) family. Full length ANXA1 binds to and activates formyl peptide receptor 2 (FPR2/ALX), whereas its *N-*terminal peptide, AC2-26 can bind and activate FPR2/ALX and formyl peptide receptor 1 (FPR1) [13]. However, the effects of ANXA1 or its associated peptides in obesity-associated inflammation are limited.

Transcriptomic analysis show increased ANXA1 expression in obese human adipose tissue and increased abundance of subcutaneous adipose tissue in old compared to young obese humans [14,15]. While rodent obese models have shown no changes in ANXA1 concentrations [16] our group has shown plasma ANXA1 to be reduced in people with obesity [17]. However, another human study reported serum ANXA1 levels to be elevated in obese diabetic patients and positively correlated with BMI, waist circumference and waist-to-hip ratio [18]. Higher levels of cleaved ANXA1 are observed in obesity, which may mediate its anti-inflammatory and pro-resolving actions locally, thus, ANXA1 may have an important pathophysiological mechanism involved in the inflammatory status observed in obesity [18]. Recent evidence suggested a role for ANXA1 protein in metabolic function, where treatment of ANXA1−/− mice with hrANXA1 restored glycaemic control [19]. 

Based on our initial observations [17], the aim of the current study was to further investigate the role of ANXA1 in obesity and its associated inflammation. The current data provides evidence from two different feasibility studies in clinical populations (1) under-going bariatric surgery and (2) patients with lipodystrophy compared to BMI matched controls. The rationale for the study was to determine whether the previous attenuation of plasma ANXA1 in human obesity [15] could be reversed by weight loss (Bariatric surgery study) and to determine whether adiposity per se was the cause of the attenuation in plasma ANXA1 or whether the inflammation associated with rising adiposity is the cause (Lipodystrophy patient data). Furthermore, the potential roles of ANXA1 in adipocytes was investigated using an in vitro cell-based model mimicking the inflammatory status that is observed during obesity. It was hypothesised that ANXA1 is positively involved in regulating processes that improve the metabolic profile of adipocytes and dampen inflammation to reduce cellular stress and the risk of developing the metabolic syndrome and its associated co-morbidities.

## 2. Materials and Methods

### 2.1. Bariatric Surgery-Subject Recruitment

Following Coventry University (P50481) and NHS (IRAS:220666) ethical approvals, participants were recruited from the general surgery pre-operative assessment clinic at Heartlands Hospital, Birmingham, UK. Fifty-two patients scheduled to undergo bariatric surgery (Sleeve Gastrectomy or Roux-en-Y gastric bypass) were screened and identified using the general surgery pre-operative assessment booking database. Thirty-four patients meeting the study inclusion criteria (male/female adults, 18–75 years old, BMI > 40 kg/m^2^, without type 2 diabetes mellitus (T2DM) or BMI > 35 kg/m^2^ if known to have T2DM) were approached at the pre-operative assessment point. Patients undergoing gastric banding, were <18 or >75 years of age, had BMI of >35 kg/m^2^, had type 1 diabetes mellitus, had mental health problems or were long term users of steroids were excluded from the study. The patients were provided with study information sheets, any questions and, apprehensions were discussed prior to enrolment. Out of the 34 patients approached, 27 patients agreed to take part in the study and provided written consent.

### 2.2. Bariatric Surgery-Patient Enrolment and Follow Up

9 patients out of the enrolled 27 patients completed the three month study protocol. Attrition in volunteers for the study included relocation to a different site for operation (1), rescheduling and/or cancellation of surgeries (4), rescheduling of follow-ups (3), loss of interest (7) and/or inability to contact patients to arrange meeting at post-opt follow ups (3).

### 2.3. Bariatric Surgery-Study Design

Patients were evaluated at baseline and three months post-surgery for weight and height, blood chemistries and plasma biomarkers. The relevant data including age, gender, past medical history, type of bariatric surgery and co-morbidities were extracted from patient notes prior to surgery. Whole blood was collected via venepuncture (10 mL; EDTA) for plasma biomarker analysis by a trained professional in-conjunction with routine blood samples required as part of the patient’s standard clinical care. 

### 2.4. Plasma Biomarker Measurement

Blood lipids including triglycerides, total cholesterol, low-density lipoprotein (LDL) and high-density lipoprotein (HDL) were assessed by local pathology lab within the hospital. Plasma biomarkers including Human C-Reactive Protein (CRP) (R&D, Minneapolis, MN, USA, #DCRP00), Human Annexin A1 (ANXA1) (Cusabio, Houston, TX, USA, #CSB-E12155h), Human Interleukin-10 (IL-10) (R&D #D1000B), Human Leptin (R&D #DLP00) and Human Adiponectin (R&D #DRP300) were quantified using enzyme linked immunosorbent assays following the manufacturers protocols. 

### 2.5. Lipodystrophy Patients

Nine lipodystrophy patient plasma samples and associated anthropometric data were supplied by Professor David Savage, Metabolic Research Laboratories, Cambridge University (Ethics Ref no. 06/Q0108/373) and were compared against BMI matched controls (*n* = 30). Plasma biomarkers CRP and Annexin A1 were quantified using the ELISA assays described in Section 2.4 following the manufacturers protocols.

### 2.6. Primary Human Adipocyte Cell Culture

Simpson Golabi Behmel Sydrome (SGBS) preadipocytes were differentiated into mature adipocytes using the revised protocol supplied with the cells from the Wabitsch Lab, (Department of Pediatrics, University of Ulm, Ulm, Germany). Briefly, SGBS cells were seeded in 6 well) using fresh growth media (DMEM/F-12 (Gibco, Thermofisher, Waltham, MA, USA, #31330-038), 10% FCS (Gibco #10270-106), 1% Antibiotic-Antimycotic (Anti-Anti) (Gibco #15240-062), 3.3mM Biotin (Fisher, Hampton, NH, USA, #144302) and 1.7 mM Pantothenate (Gibco #31330-038) and incubated for three days at 37 °C in normoxic conditions (5% CO_2_/95% air). To induce differentiation, the cells (day 0) were washed with twice with phosphate buffered saline (PBS) and incubated with serum free-QuickDiff media (DMEM/F-12, 1% Anti-Anti, 3.3 mM Biotin, 1.7 mM Pantothenate, 0.01 mg/mL Transferrin (Sigma, St. Louis, MO, USA, #T8158), 20 nM Insulin (Sigma #19278), 100 nM Cortisol (Acros, Thermofisher, Waltham, MA, USA, #352450010), 0.2 nM triiodothyronine (Sigma #T6397), 25 nM Dexamethasone (Sigma #D1756), 250 uM isobutylmethylxanthine (Acros #228420010), 2 µM Rosiglitazone (Molekula, Darlington, UK, #M34833109/Cayman#71740) for four days (37 °C/5% CO_2_/95%). Subsequently, the cells were incubated with serum free-3FC media (DMEM/F-12, 1% Anti-Anti, 3.3 mM Biotin, 1.7 mM Pantothenate, 0.01 mg/mL Transferrin, 20 nM Insulin, 100 nM Cortisol and 0.2 nM triiodothyronine) for further ten days (37 °C/5% CO_2_/95%). The media was replaced with fresh 3FC media every two to three days. At day fourteen, the cells had differentiated into mature adipocytes, with visible increase in lipid droplets. 

On day fourteen, mature adipocytes were treated with 10 µM AC2-26 peptide (Annexin A1 mimetic peptide) (Pepceuticals, Leicester, UK, #SSCU100417) or vehicle control (DMSO) and incubated in a hypoxic incubator in a humidified atmosphere containing 1% O_2_/5% CO_2_/94% N_2_ at 37 °C for 24 h to mimic adipose tissue hypoxia and generate obesogenic cell phenotype [20]. After 24 h, the cells were homogenised in Tri-Sure for mRNA expression analysis. 

### 2.7. RNA Extraction

Upon completion of the experiment, total RNA was isolated from SGBS cells using TriSure (Bioline #BIO-38032) following the manufacturers instructions. Briefly, the culture media was removed (decanted or stored at −80 °C) and cells were lysed directly in cell culture plates using 1 mL TRIsure (per 3 wells in 6 well plates) and cell were scarpered to ensure enough cell disruption. The cell homogenates were transferred into labelled DNase/RNase free Eppendorf tubes and stored at −80 °C or immediately processed. Chloroform at a ratio of 1:5 to TRIsure (e.g., 0.5 mL chloroform:1 mL TRIsure) was added to the homogenates, vigorously shaken for 15 s, and incubated for three minutes at room temperature. The homogenates were centrifuged at 12,000× *g* for 15 min at 4 °C and the aqueous phase was removed for RNA extraction. Iso-prop2nol at a ratio of 1:2 to TRIsure (e.g., 0.5 mL isopropyl alcohol:1 mL TRIsure) was added to the samples, incubated for ten minutes at room temperature, and centrifuged at 12,000× *g* for 15 min at 4 °C (followed by 2600× *g* for 30 min, if a pellet was not observed) to precipitate RNA. Finally, the pellet was washed using 75% Ethanol at 1:1 ratio to TRIsure (e.g., 1 mL 75% Ethanol:1 mL TRIsure) and centrifuged at 7500× *g* for five minutes (this step was repeated twice). The pellet was air-dried for 30 min and resuspended in 10–20 μL of DNase/RNase free water, quantified using a Nanodrop Spectrophotometer and stored at −80 °C. All the reagents used were of molecular biology grade to decrease the likelihood of contamination and RNA degradation.

### 2.8. cDNA Synthesis

SuperScript III RT enzyme kit (Invitrogen, Waltham, MA, USA, #1808044) was used to synthesise cDNA following the protocol provided by the manufacturer. Briefly, 1 μL Oligo(dT) primer (Thermofisher, Waltham, MA, USA, #18418020), 1000 ng or a maximum value of 4 μL of RNA and 1 μL 10 mM dNTP per 10 μL reaction mix were heated to 65 °C for five minutes and immediately chilled on ice, to allow the primer to anneal to the mRNA secondary structures. Then, 4 μL 1× First Strand Buffer, 1 μL 0.1 M DTT and 1 μL SuperScript III RT were added to the reaction mix and incubated at 50 °C for 60 min for cDNA synthesis followed by 70 °C for 15 min to inactivate the enzyme. The concentration of the samples was quantified using a Nanodrop Spectrophotometer and stored at −20 °C. All the reagents used were of molecular biology grade to decrease the likelihood of contamination and cDNA degradation. 

### 2.9. Real-Time qPCR

The BioRad obesity plate (Biorad, Hercules, CA, USA, #10038460) used to determine changes in mRNA/gene expression of SGBS cells following the treatments outlined above. 5 μL 2× SensiFASTTM SYBR HI-ROX Mix (Bioline, London, UK, #BIO92005) and 80 ng of cDNA per 10 μL reaction were added to each well. Additional primer assays of Resistin, Visfatin and Adipsin (*RETN* (Qiagen, Manchester, UK #QT00210483), *NAMPT* (Qiagen, Manchester, UK #QT00087920), *CFD* (Qiagen, Manchester, UK #QT00212191), respectively) were prepared. Each sample was run in triplicate. The plates were sealed, and the Bio-Rad CFX96 qPCR machine was set up with the following: activation at 95 °C (2-min cycle), denaturation at 95 °C (5 s, 40 cycles), annealing/extension at 60 °C (30 s, 40 cycles), followed by a melt curve at 65–95 °C (5 s cycle). Average Ct values were used to calculate the relative gene expression in response to AC2-26 treatment. The data obtained from the PCR reactions were normalised against the housekeeping gene (GAPDH) and analysed manually. The relative gene expression was calculated using the 2^−∆∆CT^ (Livak) method [21]. 

### 2.10. Statistical Analysis

GraphPad Prism Version 8 was used to statistically analyse the data and produce the graphs. One-tailed unpaired student’s *t*-test and one-tailed Pearson’s correlation coefficient were used to statistically analyse the data and to assess the differences in the data sets, a *p*-value of <0.05 was considered statistically significant. The number of subjects used for analysis changes due to missing data provided by the pathology lab, lack of sample due to missed appointment or outliers. Data presented is expressed as Mean ± Standard Error of the Mean (SEM).

## 3. Results

### 3.1. Plasma Annexin A1 Levels Pre and Post Bariatric Surgery

Anthropometric data and plasma samples were analysed from consenting patients undergoing routine bariatric surgery. Plasma samples were analysed for lipids and inflammatory biomarkers pre-operatively and 3 months post-operatively and correlated with ANXA1 levels. The participant characteristics of the cohort are presented in Table 1. No statistical difference was observed in average plasma concentrations of triglycerides (1.4 ± 0.3 mmol/L, *n* = 9 vs. 1.3 ± 0.1 mmol/L, *n* = 25, *p* = 0.283), total cholesterol (4.5 ± 0.4 mmol/L, *n* = 9 vs. 4.5 ± 0.1 mmol/L, *n* = 25, *p* = 0.432, HDL (1.3 ± 0.1 mmol/L, *n* = 9 vs. 1.2 ± 0.1 mmol/L, *n* = 25, *p* = 0.075) and LDL (2.5 ± 0. mmol/L, *n* = 9 vs. 2.7 ± 0.1 mmol/L, *n* = 25, *p* = 0.221) post-opt compared to pre-opt, respectively (Table 2). Weak correlations between ANXA1 and plasma triglycerides (r = 0.144, *p* = 0.236, *n* = 27), total cholesterol (r = 0.103, *p* = 0.304, *n* = 27) and LDL (r = −0.166, *p* = 0.204, *n* = 27) were observed. 

**Figure 1:**
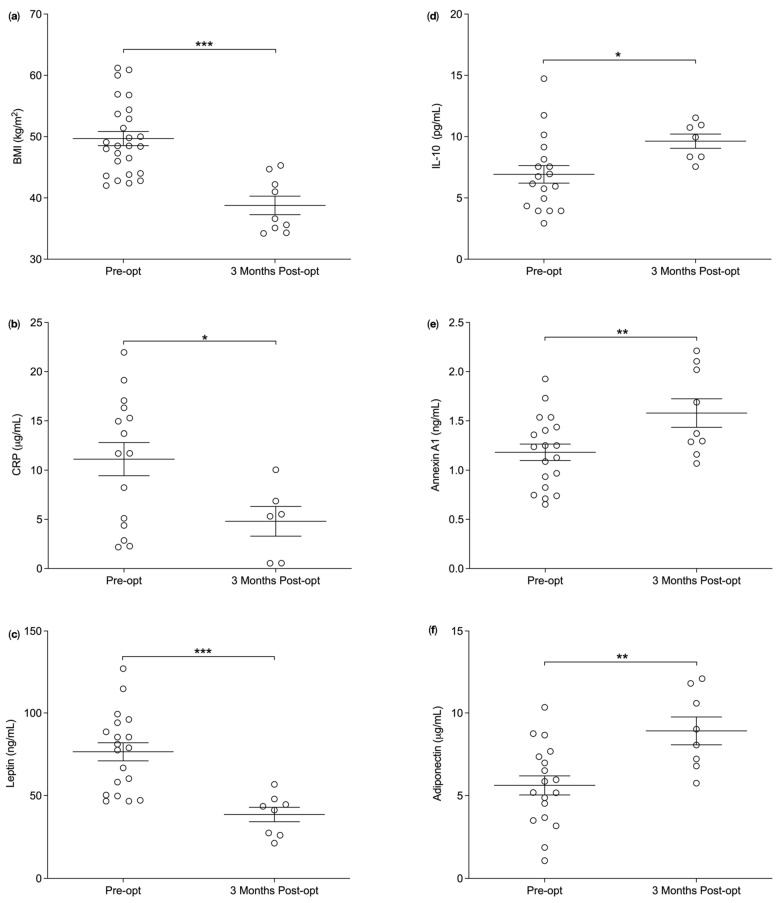
Average biomarker concentrations of participant cohort pre- and post- Bariatric surgery. (**a**) The average body mass index (BMI) (kg/m^2^) was significantly reduced at post-opt compared to pre-opt (*p* < 0.0001). (**b**) The average plasma concentration of C-reactive protein (CRP) (μg/mL) were significantly decreased at post-opt compared to pre-opt (*p* = 0.020). (**c**) The average plasma of concentration of leptin (ng/mL) were significantly decreased at post-opt compared to pre-opt (*p* = 0.0001). (**d**) The average plasma concentration of interleukin-10 (IL-10) (pg/mL) were significantly increased at post-opt compared to pre-opt (*p* = 0.018). (**e**) The average plasma concentration of annexin A1 (ng/mL) were significantly increased at post-opt compared to pre-opt (*p* = 0.009). (**f**) The average plasma concentration of adiponectin were significantly increased at post-opt compared to pre-opt (*p* = 0.002). One-tailed unpaired *t*-test was used to statistically analyse the data. Data presented as Mean ± SEM, *p* value was set at *p* < 0.05 and is denoted by * = *p* = 0.05, ** = *p* = 0.001 or *** = *p* < 0.0001. Pre-opt = Pre-operatively and Post-opt = Post-operatively.

Average BMI were significantly lower at post-opt compared to pre-opt (49.7 ± 1.2 kg/m^2^, *n* = 26 vs. 38.8 ± 1.5 kg/m^2^, *n* = 9, *p* < 0.0001) (Figure 1a). The average plasma levels of CRP 11.1 ± 1.7 µg/mL, *n* = 15 vs. 4.8 ± 1.5 µg/mL, *n* = 6, *p* = 0.0199) (Figure 1b) and Leptin (76.5 ± 5.5 ng/mL, *n* = 19 vs. 38.6 ± 4.4 ng/mL, *n* = 8, *p* = 0.0001) (Figure 1c) were significantly reduced at post-opt compared to pre-opt, suggesting decreased levels of low-grade systemic inflammation at three months post-surgery. Furthermore, the average plasma concentrations of interleukin-10 (IL-10) (6.9 ± 0.7 pg/mL, *n* = 18 vs. 9.6 ± 0.6 pg/mL, *n* = 7, *p* = 0.0181) (Figure 1d), ANXA1 (1.2 ± 0.1 ng/mL, *n* = 19 vs. 1.6 ± 0.1 ng/mL, *n* = 9, *p* = 0.009) (Figure 1e) and adiponectin (5.6 ± 0.6 µg/mL, *n* = 18 vs. 8.9 ± 0.8 µg/mL, *n* = 8, *p* = 0.0020 (Figure 1f) were significantly increased at post-opt compared to pre-opt further reiterating decreased levels of low-grade systemic inflammation following surgery. 

**Figure 2:**
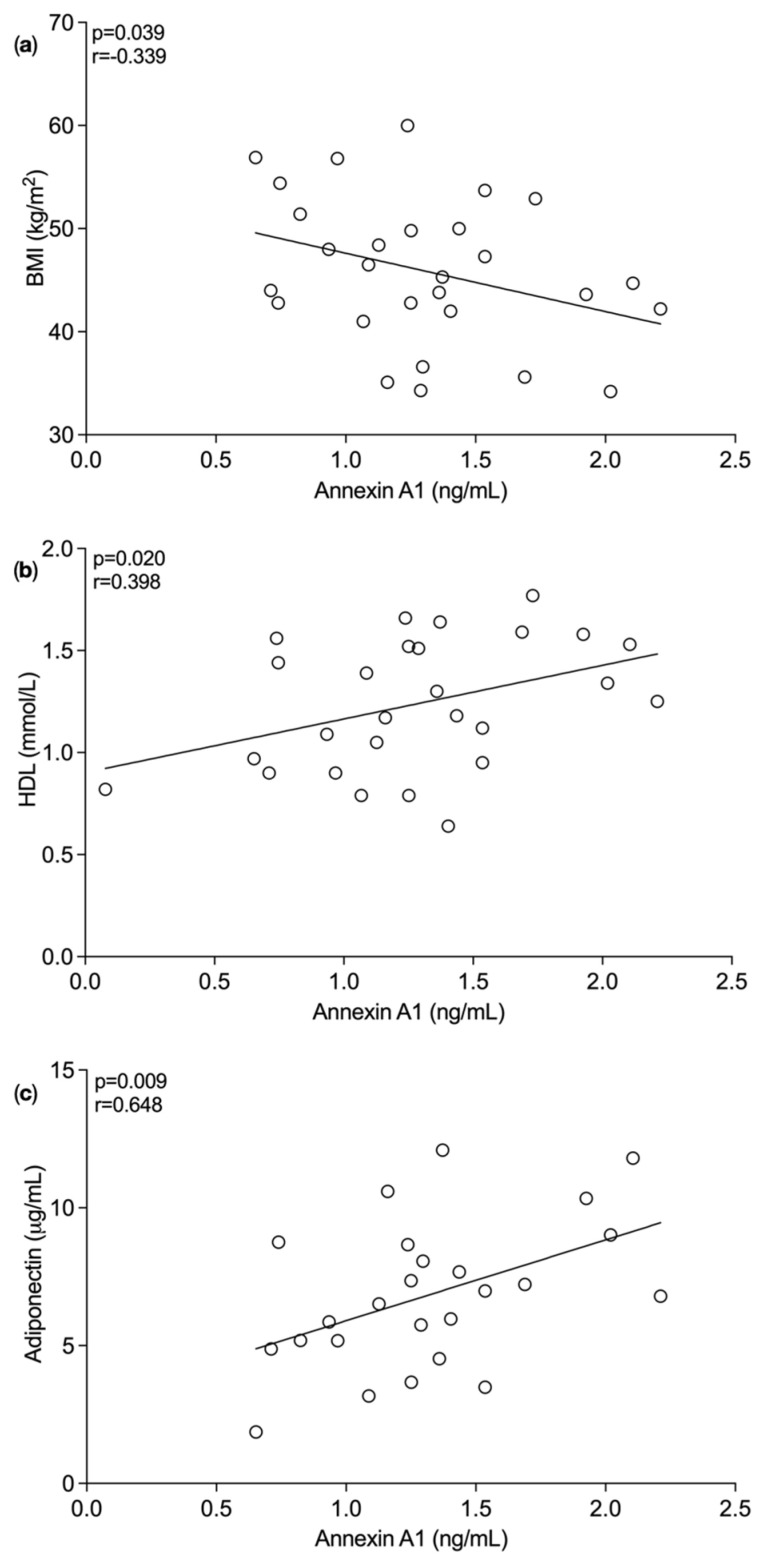
Correlation between plasma concentration of annexin A1 (ng/mL) and biomarkers in bariatric surgery patients. (**a**) Plasma concentration of Annexin A1 significantly inversely correlated with body mass index (BMI) (kg/m^2^) (*p* = 0.039, r = −0.340), (**b**) significantly positively correlated with plasma concentration of high-density lipoprotein (HDL) (mmol/L) (*p* = 0.020, r = 0.398) and (**c**) significantly positively correlated with plasma adiponectin concentration (*p* = 0.009, r = 0.468). One-tailed Pearson correlation coefficient was used to statistically analyse the data.

In line with the previous data, the average plasma concentration of ANXA1 significantly inversely correlated with BMI (*p* = 0.039, r = −0.339, *n* = 26, as shown in Figure 2a [16]. The average plasma concentration of ANXA1 significantly positively correlated with the average plasma levels of HDL (r = 0.398, *p* = 0.020, *n* = 27), as shown in Figure 2b. Further analysis showed a positive correlation between adiponectin and ANXA1 (*p* = 0.009, r = 0.468, *n* = 25) (Figure 2c). These correlations indicate plasma ANXA1 levels maybe associated with improved inflammatory status in bariatric surgery patients, suggesting a role in resolving inflammation and reducing the risk of developing co-morbidities.

### 3.2. Plasma Biomarker Levels in Lipodystrophy Patients versus BMI Matched Controls

**Figure 3:**
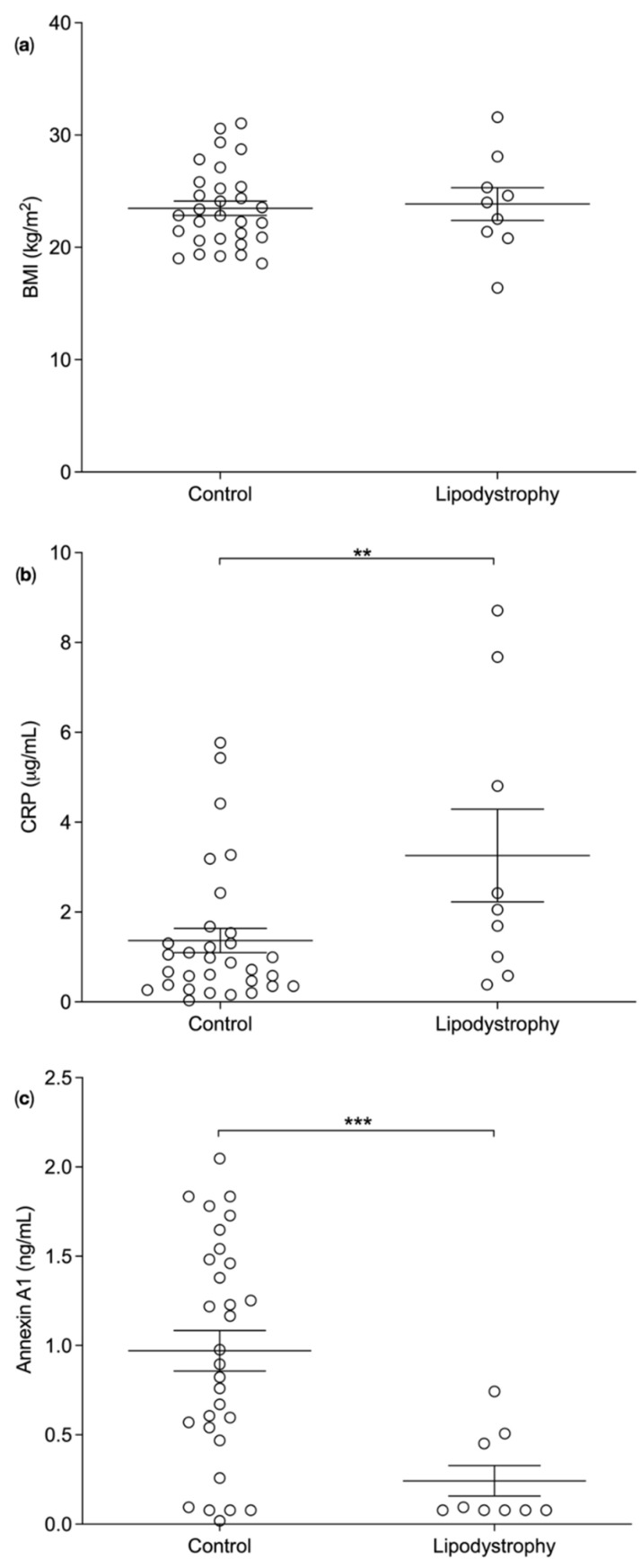
Average biomarker concentrations in lipodystrophy patients and matched controls. (**a**) No significant difference between the body mass index (BMI) (kg/m^2^) of lipodystrophy patients and control was observed (*p* = 0.324). (**b**) The average plasma concentration of C-reactive protein (CRP) (μg/mL) were significantly increased (*p* = 0.0074) and (**c**) the average plasma concentration of annexin A1 (ng/mL) were significantly reduced (*p* = 0.0008) in lipodystrophy patients compared to controls. One-tailed unpaired *t*-test was used to statistically analyse the data. Data presented as Mean ± SEM, *p* value was set as *p* < 0.05 and is denoted by ** = *p* = 0.001 or *** = *p* < 0.0001.

Anthropometric data and plasma samples of 9 consented lipodystrophy patients and 31 consented BMI matched controls were analysed. Plasma samples were analysed for plasma inflammatory biomarkers and correlated with plasma ANXA1 levels. BMI of lipodystrophy patients and matched control were not different (23.9 ± 1.5 kg/m^2^, *n* = 9 vs. 23.2 ± 0.6 kg/m^2^, *n* = 29, *p* = 0.324) (Figure 3a). As expected, CRP concentrations were significantly elevated in lipodystrophy patients compared to control (3.3 ± 1.0 µg/mL, *n* = 9 vs. 1.4 ± 0.3 µg/mL, *n* = 31, *p* = 0.0074) (Figure 3b) [22]. Interestingly, the average plasma levels of ANXA1 were significantly lower in lipodystrophy patients compared to control (0.2 ± 0.1 ng/mL, *n* = 9 vs. 0.97 ± 0.1 ng/mL, *n* = 30, *p* = 0.008) (Figure 3c).

### 3.3. Cell Culture Experiments Using Human Primary Adipocytes (SGBS Cells)

**Figure 4:**
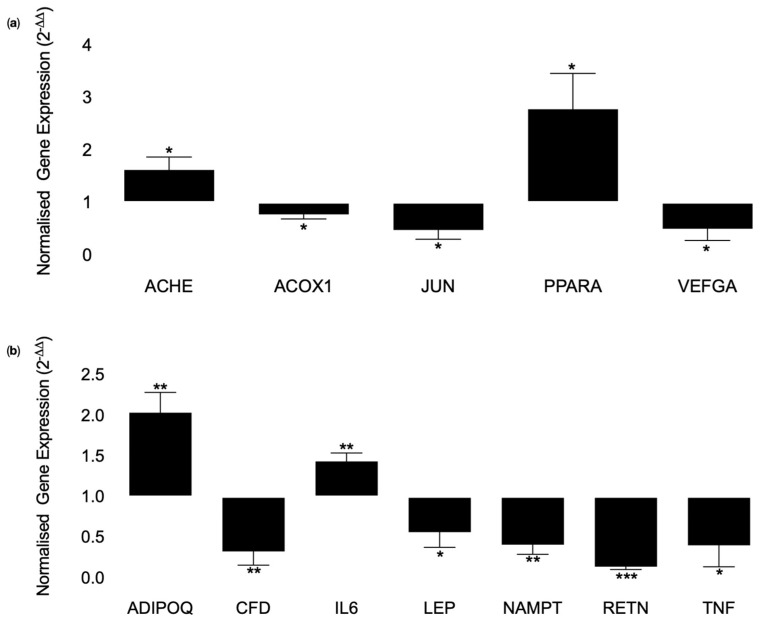
mRNA expression of genes known to be dysregulated in obesity in response to acute AC2-26 treatment using a human adipocyte model mimicking the inflammatory status observed in obesity. Simpson Golabi Behmel Syndrome (SGBS) cells were differentiated into mature adipocytes and treated with 10 μM AC2-26 or vehicle control and incubated in hypoxia (1%O_2_) environment for 24 h. mRNA was extracted using Trisure and cDNA was synthesized. 80 ng of cDNA was used in SYBR green mediated quantitative PCR. Ct values were obtained and used to calculate normalized gene expression using the Livak 2^−^^ΔΔ^^CT^ equation. One-tailed unpaired student’s *t*-test was used to statistically analyse the data. (**a**) Genes involve in adipocyte development; *ACHE* (*p* = 0.0317, *n* = 3) and *PPARA* (0.0303, *n* = 3) were significantly upregulated, whereas, *ACOX1* (*p* = 0.0303, *n* = 3), *JUN* (*p* = 0.02006, *n* = 3) and *VEGFA* (*p* = 0.0449, *n* = 3) were significantly downregulated in response to acute AC2-26 treatment compared to vehicle control. (**b**) Genes involved in inflammation; *ADIPOQ* (*p* = 0.0073, *n* = 3) and *IL-6* (*p* = 0.0072, *n* = 3) were significantly upregulated and *CFD* (*p* = 0.0096, *n* = 3), *LEP* (*p* = 0.040, *n* = 3), *NAMPT* (*p* = 0.0039, *n* = 3), *RETN* (*p* < 0.0001, *n* = 3) and *TNF* (*p* = 0.0429, *n* = 3) were significantly downregulated in response to acute AC2-26 treatment compared to vehicle control. Vehicle control normalized to 1. Vehicle control = contains DMSO. Data presented as Mean ± SEM, *p* value was set as *p* < 0.05 and is denoted by * = *p* = 0.005, ** = *p* = 0.001 or *** = *p* < 0.0001 vs. 1.

The mRNA expression of genes involved in processes that regulate the metabolic and inflammatory profile in adipocytes in response to acute treatment with ANXA1 peptide; AC2-26 was investigated. The Database for Annotation, Visualization, and Integrated Discovery (DAVID) bioinformatics resource 6.7 (https://david-d.ncifcrf.gov/home.jsp (accessed on 24 April 2022)) was used to categorise the genes using the Kyoto Encyclopaedia of genes and genomes (KEGG) database into biological pathways for analysis. Genes involved in adipocyte development and inflammation were extracted and analysed in more detail. The mRNA expression of *acetylcholinesterase* (*ACHE*) (1.6 ± 0.2-fold, *p* = 0.0317, *n* = 3) and peroxisome proliferator activated receptor alpha (*PPARA*) (2.8 ± 0.7-fold, *p* = 0.0303, *n* = 3), were significantly upregulated and acyl-coA oxidase 1 (*ACOX1*) (0.78 ± 0.1-fold, *p* = 0.0303, *n* = 3), *jun* proto-oncogene (*JUN*) (0.5 ± 0.2-fold, *p* = 0.0206, *n* = 3), vascular endothelial growth factor A (*VEFGA*) (0.5 ± 0.2-fold, *p* = 0.0449, *n* = 3), were significantly downregulated in response to acute AC2-26 treatment compared to control, as shown in Figure 4a. mRNA expression of adiponectin, C1Q and collagen domain containing (*ADIPOQ*) (2.0 ± 0.3-fold, *p* = 0.0073, *n* = 3) and interleukin-6 (*IL-6*) (1.4 ± 0.1-fold, *p* = 0.0072, *n* = 3) were statistically significantly upregulated and complement factor D (*CFD*) (0.3 ± 0.2-fold, *p* = 0.0096, *n* = 3), leptin (*LEP*) (0.6 ± 0.2-fold, *p* = 0.0400, *n* = 3), nicotinamide phosphoribosyltransferase (*NAMPT*) (0.4 ± 0.1-fold, *p* = 0.0039, *n* = 3), resistin (*RETN*) (0.1 ± 0.03-fold, *p* < 0.0001, *n* = 3) and tumour necrosis factor (*TNF*) (0.4 ± 0.3-fold, *p* = 0.0429, *n* = 3) were statistically down-regulated in response to acute AC2-26 treatment compared to control, as shown in Figure 4b. 

## 4. Discussion

The aim of the current study was to investigate the role of ANXA1 in obesity and associated inflammation using relevant human and cell culture models. Plasma ANXA1 levels were significantly increased three months-post surgery in bariatric surgery patients and in line with the results of our previous study, inversely correlated with BMI [16]. Plasma HDL levels increased post-surgery and positively correlated with ANXA1. Plasma biomarkers (CRP and leptin) associated with exacerbated inflammatory status were decreased and inversely correlated with plasma ANXA1 levels. Likewise, plasma biomarkers IL-10 and adiponectin associated with improved inflammatory status were increased post-surgery and directly correlated with plasma ANXA1 levels. 

Approximately, 60–70% of individuals with obesity have abnormal lipid profiles (dyslipidaemia), including increased circulating LDL levels, increased circulating triglycerides and decreased circulating HDL, thereby, significantly increasing the risk of cardiovascular diseases in these individuals [23]. Bariatric surgery induced weight loss is associated with reduced circulating triglyceride levels, reduced LDL levels and increased circulating HDL levels, thereby significantly reversing dyslipidaemia [24,25,26]. This pattern was also observed in the current study, however, statistical power was not reached, possibly owing to small sample size as the retention rate throughout the study was relatively low (33%). Furthermore, the results of the current study are the first to show a significant positive correlation between plasma ANXA1 and plasma HDL levels and inverse correlation between plasma ANXA1 and plasma LDL levels in bariatric surgery patients. Both ANXA1 and HDL mediate cardioprotective effects due to their anti-oxidative, anti-inflammatory, and anti-atherogenic properties [27]. Increased expression of ANXA1 in atherosclerotic plaques in carotid stenosis patients without clinical symptoms and deletion of FPR2/ALX receptor or ANXA1 in Apolipoprotein E (*APO−/−*) high fat diet (HFD) fed mice enhanced atherosclerotic lesion formation, suggesting ANXA1 may be involved in resolving and/or attenuating progression of plaque formation [28,29]. However, FPR2/ALX expression levels are upregulated in human carotid atherosclerotic lesions compared to healthy vessels and is suggested to promote disease progression and increase plaque stability [30]. A recent study reported a novel anti-inflammatory mechanism of HDL via modulation of the expression of ANXA1 in vascular endothelial cells in a dose dependent manner [27]. The increase was associated with inhibition of cell surface adhesion molecules and secretion of chemotactic factor, inhibiting monocyte adhesion, thereby, attenuating formation of atherosclerotic lesions [27]. 

Obesity associated inflammation is initiated within the adipose tissue with elevated macrophage infiltration into adipose depots and plasma expression of pro-inflammatory cytokines and adipokines. These pro-inflammatory cytokines and adipokines enter the circulation and cause systemic inflammation and contribute to the onset of obesity associated comorbidities such as cardiovascular disease and T2DM [31]. IL-10 is a pleiotropic anti-inflammatory cytokine involved in immunoregulation and inflammation. It is considered to play a protective role in human metabolism, as it is shown to provide protection against endothelial dysfunction during T2DM [32]. Plasma IL-10 levels are significantly increased after weight loss induced by lifestyle changes and bariatric surgery, and significantly correlate with plasma adiponectin levels [33,34,35]. Furthermore, recombinant ANXA1 stimulates IL-10 secretion from macrophages primed with liposaccharide in a dose dependent manner, suggesting ANXA1 may partly mediate its anti-inflammatory actions by modulating the secretion of IL-10 [36]. The changes in plasma adiponectin and leptin concentrations in the current study may reflect decrease in adipose tissue mass, increase in insulin sensitivity and reduction in inflammation. In the current study, plasma adiponectin levels directly correlated with plasma ANXA1 levels, which may reflect an insulin sensitising role for ANXA1. Furthermore, direct correlation with adiponectin levels suggests, plasma ANXA1 levels may be associated with adiposity levels, as suggested previously [16]. 

**Figure 5:**
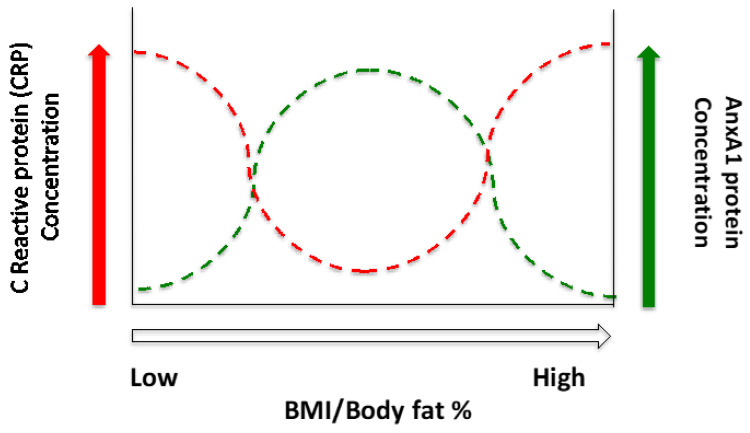
Proposed relationship between CRP and ANXA1 plasma concentrations and BMI/body fat (%) based on the current and previous research.

The anti-inflammatory actions of ANXA1 may be compromised by obesity which may alter ANXA1’s autocrine, paracrine effects on adipose tissue to resolve inflammation. However, the current data would suggest that this effect may be reversed in response to significant weight loss, therefore, aiding in reducing systemic inflammation and the risk of developing obesity associated co-morbidities. However, plasma ANXA1 levels were also significantly decreased in patients with lipodystrophy compared controls and inversely correlated with BMI, in a similar manner that is observed in obese individuals, suggesting the degree of adiposity may not be the principal cause of attenuated plasma ANXA1 levels, despite these factors being negatively correlated [16]. The results of the current study indicate that plasma ANXA1 levels are negatively correlated with pro-inflammatory cytokines (CRP), as previously described [16], however the results from the lipodystrophy patients suggests that this is not mediated merely by body morphology. Lipodystrophies are a group of heterogenous diseases characterised by generalised or partial loss of adipose tissue. Congenital generalised lipodystrophies are characterised by the loss of adipose tissue caused my genetic mutations and are further divided into 4 subtypes based on the gene involved, whereas, acquired generalised lipodystrophies are characterised by general loss of adipose tissue caused by inflammation and autoimmune diseases [37,38]. The attenuated ANXA1 levels observed in lipodystrophy patients with low adiposity and in obese participants with high adiposity may therefore result from an increased systemic inflammatory environment where the balance of inflammation is tipped in favour of pro-inflammatory factors (Figure 5). 

The origin of inflammation in lipodystrophy varies amongst the different subtypes and may depend on the aetiology. Inflammation in lipodystrophy patients is thought to play a differential role to that of obesity induced inflammation. For example, increased infiltration of classically activated macrophages (M1) into the adipose tissue and increased production of TNFα and IL-6 are hypothesized to interfere with the normal activity of insulin receptors and contribute to the development of insulin resistance in obesity [39]. Although, macrophage infiltration is also increased in lipodystrophic adipose tissue, their phenotype and behaviour (Combination of M1 and M2) is very different. They are believed to be involved in tissue remodelling and repair due to increased apoptosis and not in the pathogenesis of insulin resistance [40]. Furthermore, other researchers have demonstrated that anti-inflammatory drugs known to decrease inflammation and improve hyperglycaemia by lowering glucose and insulin levels in human obese and diabetic individuals are ineffective in aP2-nSREBP-1C transgenic mouse model (resembles congenital generalised lipodystrophy in humans) [40]. Instead, the altered synthesis and secretion of adipokines is hypothesised to induce lipodystrophy associated metabolic abnormalities [21]. 

The discrepancy between the circulating levels of ANXA1 in our two human studies compared to the work of Pietrani and colleagues [18] is difficult to explain without further experimental work. Differences in ANXA1 protein levels between plasma and serum may be the cause of the discrepancy, although this would require further work. The source of plasma ANXA1 protein or the reason for its attenuation in obesity is not currently understood. However, there is evidence from other clinical conditions such as patients with rheumatoid arthritis that in an environment of chronically elevated levels of inflammation ANXA1 protein levels are suppressed compared to controls [41] and that ANXA1 response to exogenous glucocorticoids is reduced, suggesting that under certain conditions the normal regulation of ANXA1 by glucocorticoids may be disrupted. The is also evidence for ANXA1 auto-antibody production in rheumatoid arthritis and other chronic inflammatory diseases such as Lupus (Systemic Lupus Erythematosus-SLE) patients [42] which may account for the reduction in biologically available plasma ANXA1 protein. Additionally, a number of previous studies using animal models explored the mechanistic aspects have demonstrated that ANXA1 is involved in controlling/suppressing the release of adipocyte IL-6 [43] and IL-6 and TNF release from macrophages [44]. These studies identify possible mechanisms by which reductions in the concentration of ANXA1 protein may lead to increased expression of inflammatory cytokines, although this does not identify what cause of the reduction in plasma ANXA1concentration. It is of interest for future studies to investigate the effects of systemic inflammation on the expression and regulation of ANXA1 in obesity. 

Additionally, the in vitro results from the current study indicate that ANXA1 may be involved in regulating genes in metabolic processes in adipocytes, thereby, affecting the local and peripheral inflammatory status. Interestingly, mRNA expression of *ACOX1* was significantly downregulated and *PPARA* was significantly upregulated in response to AC2-26 treatment, in the current study. Inhibition of *ACOX1* increases hepatic mitochondrial fatty acid oxidation, *PPARα* expression, decreases body weight gain, circulating triglycerides and insulin levels in high fat diet fed rats [45]. Furthermore, activation of *PPARα* attenuates obesity, increases fatty acid oxidation, restores glucose homeostasis by preventing the development of obesity induced insulin resistance and supresses obesity-induced increase in inflammatory cytokines such as, TNFα by inhibiting the nuclear factor kappa β signalling pathway and reduces macrophage derived inflammation [46,47,48,49,50]. Furthermore, activation of *PPARα* prevents adipocyte hypertrophy by increasing energy expenditure via inducing pathways involved in fatty acid consumption [51]. Trans-differentiation from white to brown adipocytes or brown-like adipocytes is suggested to have significant implications in the treatment of obesity and associated metabolic disorders [52]. ANXA1 may induce this transformation or decrease adipocyte hypertrophy by regulating *PPARα* activity, therefore, it would be of significant interest to further understand the implications of *PPARα* activation by AC2-26 and/or ANXA1 in an obesogenic adipocyte model. 

In the current study using SGBS cells, the mRNA expression of the pro-inflammatory adipokines and cytokines; *LEP*, *NAMPT*, *RETN*, *CFD* and *TNFα* and anti-inflammatory adipokines; *ADIPOQ* was significantly downregulated and upregulated respectively in response to acute AC2-26 treatment. Previous studies show positive correlations between Leptin, Visfatin, Resistin and Adipsin and circulating inflammatory markers such as IL-6 and CRP in individuals suffering from obesity [53,54,55,56,57,58]. Conversely, adiponectin negatively correlates with obesity and its overexpression improves glucose metabolism, reduces macrophage numbers, decreases TNFα and improves vascularisation in *ob*/*ob* mice [59]. TNFα is increased in the adipose tissue of human and mice models of obesity and T2DM [60,61]. In addition, TNFα contributes to dysregulated secretion of adipokines, as obese subjects overproduce adipokines due to hyperactivation of nuclear factor kappa light chain enhancer of the activated B cells (NF-κB) pathway [62]. Overexpression of adipokines and cytokines in adipocytes from obese subjects can be prevented by inhibiting NF-κB activity. Therefore, ANXA1 may reduce adipokine dysregulation and reduce inflammation as it mediates its activity by binding to, and inhibiting NF-κB activity, enhancing apoptosis and inhibiting cell growth [63]. Interestingly, the mRNA expression of *IL-6* was upregulated in response to acute AC2-26 treatment. IL-6 induces the expression of ANXA1, which may act in an autocrine and paracrine manner to regulate the expression of IL-6 in a positive feedback loop [64]. The imbalance between the pro-inflammatory and anti-inflammatory mediators is suggested to result in insulin resistance and development of metabolic syndrome in obese individuals [62]. Mice lacking IL-6 display obesity, hepatosteatosis, liver inflammation and insulin resistance compared to wildtype mice on a standard chow diet, with increased glucose intolerance and insulin resistance in mice fed with a high fat diet compared to controls [61,65]. These data may therefore reflect some of the direct and indirect anti-inflammatory and insulin sensitising roles of ANXA1 via its interactions with other anti-inflammatory mediators. 

Jun proteins play a central role in regulating transcription of genes that are involved in development, differentiation and inflammation, and modulate the response to acute cellular insults such as oxidative stress and DNA damage [66,67,68,69,70]. Jun-D is expressed in differentiated 3T3-L1 *murine* adipocytes, where it plays an important role for the development and/or maintenance of adipocyte phenotype [71]. The mRNA expression of *JUN* was significantly downregulated in response to acute AC2-26 treatment in the current study, suggesting a role of ANXA1 in regulating adipogenesis. Vascular endothelial growth factor α (VEGFα) is a key regulator of angiogenesis in the adipose tissue and is regulated by exercise, hypoxia, insulin, cytokines, and growth factors [72]. Poor vascularisation during adipose tissue proliferation causes fibrosis, local tissue inflammation and insulin resistance [73]. VEGFα is secreted by adipocytes and acts as an autoregulatory mechanism for angiogenesis in the adipose tissue [72]. Healthy expansion of the adipose tissue occurs when it is fully vascularised, however, adipocytes frequently fail to mount a proper response to local hypoxia and do not produce enough VEGFα [73,74]. Therefore, adipose cell hypertrophy caused by obesity is associated with non-vascularised adipose tissue leading to hypoxia, fibrosis, inflammation, ectopic fat deposition, macrophage infiltration and insulin resistance [72,75]. In support of this mechanism, the overexpression of VEGFα in the adipose tissue increases the number and size of blood vessels to protect against high fat diet induced hypoxia and improves whole body insulin sensitivity and glucose tolerance [76]. Therefore, lack of sufficient blood supply to the adipose tissue is thought to lead to an earlier occurrence of hypoxia, fibroplasia, systemic insulin resistance as lipids are stored is forced to ectopic sites [31]. Therefore, in line with this observation, ANXA1 may modify or fine tune adipose tissue expansion via *VEGFα* as mRNA expression of *VEGFA* was significantly downregulated in response to acute AC2-26 treatment, in the current study. The parasympathetic neurotransmitter, acetylcholine is believed to have the ability to block inflammation by inhibiting synthesis and release of cytokines, and could serve as a biomarker of low-grade systemic inflammation as observed in obesity [77,78,79]. The mRNA expression of *ACHE* was significantly upregulated in response to acute AC2-26 treatment, in the current study. Previous studies have suggested impaired sympathetic/parasympathetic response and reduced cholinergic blockade may be responsible for the inflammation and adverse metabolic sequelae observed in obesity [80]. However, short-term cholinergic blockade with atropine; a competitive antagonist of the acetylcholine receptor enhanced insulin sensitivity in both human lean and abdominal obese subjects [81].

The current data indicate that plasma ANXA1 concentrations are significantly increased following rapid weight loss following bariatric surgery and therefore may be recoverable over time with further weight loss. The implications for human health of lower levels of plasma ANXA1 have not been evaluated at this time. Furthermore, the data presented from lipodystrophy patients suggest that plasma ANXA1 levels are not related to morphology per se, but are correlated with a pro-inflammatory environment. The clinical significance of the current data is unknown at this time, although both clinical studies described in the current manuscript would indicate that lower plasma concentrations of ANXA1 protein are associated with higher pro-inflammatory status and poorer health outcomes. Conversely, the significant increase in plasma concentrations of ANXA1 protein in the bariatric surgery group 3 months post-surgery correlate with improved health outcomes described in previously published literature [82], indicating that higher plasma concentrations of ANXA1 are related to better metabolic health. The major limitations of the bariatric surgery study was a small follow up cohort due to a high attrition rate and a predominantly female patient gender bias. Follow up studies involving lipodystrophy patients would benefit from a larger cohort and more detailed characterisation of the different lipodystrophy genotypes.

Furthermore, the results of this study describe evidence for two different but overlapping functions of ANXA1. The first of which is produced locally within the adipose tissue depots and may have a role in regulating adipogenesis and reducing adipocyte hypertrophy to prevent adipocyte dysfunction, local inflammation and development of insulin resistance and secondly peripheral circulating levels (the source of which is still unknown) have been demonstrated to negatively correlate with inflammatory biomarkers. Further investigations are required to understand the role of plasma ANXA1 protein in obesity and determine whether it has a therapeutic role in obesity management. 

## 5. Conclusions

Peripherally, ANXA1 may act in an endocrine manner to regulate inflammatory biomarkers to dampen inflammation, regulate insulin secretion and improve the metabolic profile to reduce the risk of developing obesity associated co-morbidities. However, this effect may be overshadowed by the continued increase in systemic inflammation associated with rapid adipose tissue expansion.

## Figures and Tables

**Table 1:** Participant characteristics at pre- and post- bariatric surgery. Pre-opt = Pre-operatively and Post-opt = Post-operatively.

Patient Characteristics-Bariatric Surgery Study			
		Pre-Opt	Post-Opt
**Number of participants**		*n* = 26	*n* = 11
**Gender**			
	Males	4	0
	Females	22	11
**Age, years**			
		48 ± 1.9	51 ± 2.7
**Type of Surgery**			
	Sleeve Gastrectomy	13	5
	Roux-en-Y Gastric Bypass	13	6
**Type 2 diabetes mellitus**			
	Diabetics	10	4
	Non-Diabetics	16	7

**Table 2:** Anthropometric measures and lipid profiles of participants at pre- and post- bariatric surgery.

Anthropometric Measures and LIPID PROFILES			
		Pre-Opt	Post-Opt
	Weight (kg)	131.5 ± 6.4 (*n* = 26)	105.9 ± 5.3 * (*n* = 9)
**Lipid Profiles**			
	Triglycerides (mmol/L)	1.3 ± 0.1 (*n* = 25)	1.4 ± 0.3 (*n* = 9)
	Total Cholesterol (mmol/L)	4.5 ± 0.1 (*n* = 25)	4.5 ± 0.4 (*n* = 9)
	HDL (mmol/L)	1.2 ± 0.1 (*n* = 25)	1.3 ± 0.1 (*n* = 8)
	LDL (mmol/L)	2.7 ± 0.1 (*n* = 25)	2.5 ± 0.2 (*n* = 9)

The anthropometric measures and lipid profiles were analysed in participants undergoing bariatric surgery at pre- and 3 months post-opt. One-tailed unpaired *t*-test was used to statistically analyse the data. Data presented as Mean±SEM. *p* value was set at *p* < 0.05 and is denoted by * = *p* = 0.05. Pre-opt = Pre-operatively, Post-opt = Post-operatively, BMI = Body Mass Index, HDL = High density lipoprotein and LDL = Low density lipoprotein.
